# Novel use of the Nintendo Wii board as a measure of reaction time: a study of reproducibility in older and younger adults

**DOI:** 10.1186/s12877-015-0080-6

**Published:** 2015-07-10

**Authors:** Martin Gronbech Jorgensen, Sentha Paramanathan, Jesper Ryg, Tahir Masud, Stig Andersen

**Affiliations:** Department of Geriatrics, Aalborg University Hospital, Hobrovej 18-22, DK-9000 Aalborg, Denmark; Department of Geriatrics, Odense University Hospital, Odense, Denmark; Institute of Clinical Research, University of Southern Denmark, Odense, Denmark; Health care for Older People, Nottingham University Hospital NHS Trust, Nottingham, Notts UK; Department of Clinical Medicine, Aalborg University, Aalborg, Denmark

**Keywords:** Reaction time, Older adults, Young adults, Reproducibility, Nintendo wii board

## Abstract

**Background:**

Reaction time (RT) has been associated with falls in older adults, but is not routinely tested in clinical practice. A simple, portable, inexpensive and reliable method for measuring RT is desirable for clinical settings. We therefore developed a custom software, which utilizes the portable and low-cost standard Nintendo Wii board (NWB) to record RT. The aims in the study were to (1) explore if the test could differentiate old and young adults, and (2) to study learning effects between test-sessions, and (3) to examine reproducibility.

**Methods:**

A young (*n* = 25, age 20–35 years, mean BMI of 22.6) and an old (*n* = 25, age ≥65 years, mean BMI of 26.3) study-population were enrolled in this within- and between-day reproducibility study. A standard NWB was used along with the custom software to obtain RT from participants in milliseconds. A mixed effect model was initially used to explore systematic differences associated with age, and test-session. Reproducibility was then expressed by Intraclass Correlation Coefficients (ICC), Coefficient of Variance (CV), and Typical Error (TE).

**Results:**

The RT tests was able to differentiate the old group from the young group in both the upper extremity test (*p* < 0.001; −170.7 ms (95%CI −209.4;-132.0)) and the lower extremity test (*p* < 0.001; −224.3 ms (95%CI −274.6;-173.9)). Moreover, the mixed effect model showed no significant learning effect between sessions with exception of the lower extremity test between session one and three for the young group (−35,5 ms; 4.6 %; *p* = 0.02). A good within- and between-day reproducibility (ICC: 0.76-0.87; CV: 8.5-12.9; TE: 45.7-95.1 ms) was achieved for both the upper and lower extremity test with the fastest of three trials in both groups.

**Conclusion:**

A low-cost and portable reaction test utilizing a standard Nintendo wii board showed good reproducibility, no or little systematic learning effects across test-sessions, and could differentiate between young and older adults in both upper and lower extremity tests.

## Background

Fall accidents within the rapidly growing ageing population [1] is a major concern worldwide due to serious consequences such as increased morbidity, decreased functional levels, admission to long-term care facilities, and higher mortality [2, 3]. Many reports have associated fall accidents in older adults with an increased reaction time (RT) in either the upper or lower extremities [4–9] but is not routinely tested in clinical practice. Lord *et al.* [5] performed a prospective study on 341 community-dwelling women (+65 years of age) and found a strong association between fallers and increased lower limb RT compared to the non-fallers. Another prospective study [6] found that upper and lower-extremity RT along with other physiological, cognitive and medical factors could discriminate between fallers and nonfallers. These studies along with others have paved the way for the physiological profile approach [10], which uses both upper and lower extremity RT when determining the fall risk of older adults. Finally, several researchers have found RT measures to be responsive to exercise interventions in older adults [11–13], making the RT measures relevant in clinical practice. A common protocol for assessing RT is to measure the time between the presentation of a light stimulus and subsequently hitting a response button [14] testing either the upper [15], the lower [8] or both extremities [16]. RT has been tested extensively in both athletic- (i.e. soccer [17], racecar [18], lacrosse [19]) and nonathletic populations (i.e. single fallers vs. recurrent fallers [8], community-dwelling older adults [16], sport science students [20]), and various age groups [16, 21–23]. In most cases these studies have used expensive laboratory equipment [8, 16–21], which prevents wide application of RT testing. Occasionally a fast, simple and inexpensive method for measuring RT has been tested, but subsequently reported non-reliability [15]. This highlights the need for a reliable, inexpensive, widely available, and portable system for evaluating RT. The Nintendo Wii Board (NWB) could satisfy these needs as it measures 50 cm × 30 cm × 5 cm, weighs 4.5 kg, has a price tag around 100$, and currently over 43 million copies of the NWB have been sold worldwide. Moreover, the NWB has currently been used in other scientific studies along with “off the shelf” software for exercise interventions [24, 25], evaluation of postural balance [26], prediction of falls [27], and custom software for postural balance evaluation [28] in children.

However, to our knowledge no previous studies have explored the NWB for evaluating upper- and lower-extremity RT. Thus, we developed a software application, which utilizes the force transducers of the NWB to record RT in the both the upper and lower extremities. Thus, the aim of this study was (1) to explore if RT could differentiate between older and younger adults, and (2) to determine if a learning effect existed between test-sessions, and (3) finally to examine reproducibility of the RT test.

## Methods

### Study population

We recruited participants in two age groups. Participants in the younger group (20–35 years of age) were recruited from the campus of Aalborg University, Denmark and participants in the older group (≥65 years of age) from senior citizen clubs and organizations in the Aalborg area (Table 1). In both groups participants were excluded if they had any acute illness within the previous 3 weeks, neurological disease (such as dementia, Parkinson), visual impairment (Snellen score <3/60), were taking medication (psychotropic, hypnotics or anti-depressive) that could influence RT, or had orthopedic surgery within the previous 6 months.

**Table 1:** Participants characteristics

Age group	Women (%)	Age (yr.)	Height (cm)	Weight (kg)	BMI	Medicine (no.)	PAL
Young (*n* = 25)	48	24.7 ± 3.0	174.6 ± 7.5	69.3 ± 9.3	22.6 ± 2.3	0	9.8 ± 3.7
Old (*n* = 25)	68	74.2 ± 6.1	168.3 ± 4.6	74.8 ± 13.4	26.3 ± 4.7	2.75 ± 5.0	5.3 ± 4.1

Characteristics of the participants presented as mean ± SD. PAL denotes: Physical Activity Level in hours per week

### Study design

**Fig. 1:**
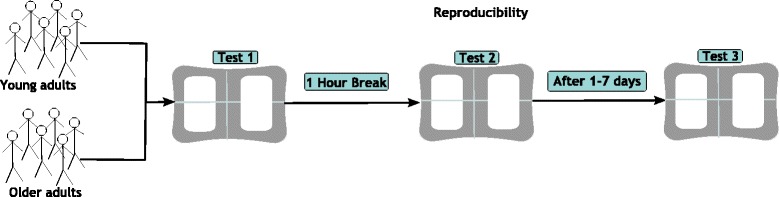
Overview of the study design

The study was designed, performed, and analyzed according to guidelines for reporting reliability and agreement studies (GRRAS) [29]. A within- and between-day design was applied using a single rater (intra-rater). Within-day reproducibility was explored using a one-hour break between sessions, and between-day reproducibility tested with 1–7 days between sessions (Fig. 1).

### Test procedures

**Fig. 2:**
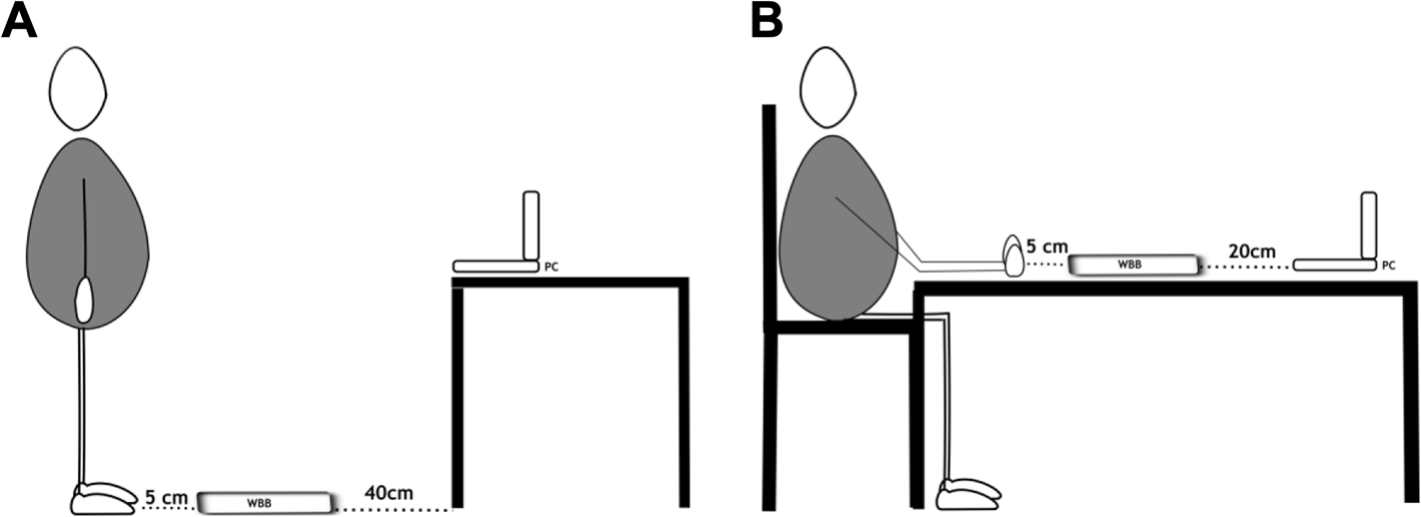
Illustration of the reaction test setup for (**a**) lower extremities and (**b**) the upper extremities

To collect data from the NWB a laptop computer (Lenovo Yoga Pro, Windows 8) was connected using a bluetooth HID wireless protocol and imported into a custom program written in C#. The evaluation of the RT in the lower (FOOT) and upper (HAND) extremity was carried out by performing a series of step- and hammering-tests on the NWB. In the stepping test, (Fig. 2a) participants were positioned in bilateral weight bearing position directly in front of the NWB with approximately 100 cm to the screen of a laptop computer. For the hammering test, (Fig. 2b) participants were seated on a standard chair with the NWB in front of their fists with the laptop screen approximately 80 cm away from their eyes. In both tests, participants were instructed to react as fast as possible according to a visual stimulus displayed on the computer. The visual stimulus was presented as a green light at a random time (between 1 and 4 s) and side (either left or right) on the computer screen. The internal timer would be stopped when the appropriate side (according to the visual clue) of the NWB was hit. For each test, the operator had to restart the test immediately after the previous test, resulting in random inter-trial intervals.

The elapsed time given in milliseconds (RT) from the visual stimulus was displayed until the participant responded by stepping or hammering on the NWB was then logged and stored in a database on the computer for further analysis. At each session (1, 2 and 3) participants completed 10 consecutive step (5 left and 5 right in random order) and 10 consecutive hammering-tests (5 left and 5 right in random order) totaling 60 trials per participant in the whole study.

### Statistical analysis

All statistical analyses were performed using SPSS (version 21, IBM, New York, USA). A statistician at the department of statistics, Aalborg University Hospital performed and validated the statistical models used in this study. Reaction times were determined using the developed software and expressed as mean ± SD.

Preliminary analysis of the data focused on systematic differences associated with age and session (learning effects), and a mixed effects model was applied. This model used subjects as a random effect and age, and session as fixed effects. Reproducibility was afterwards expressed for within-day and between-day by Interclass Correlation Coefficients (ICC), Coefficient of Variance (CV), and Typical Error (TE) [29]. In addition, we reported different means and fastest values of the recordings in order to give the reader added transparency of the reproducibility. ICC was chosen to assess relative reliability and determined as between-subject variance versus total variance, and was interpreted using the following criteria: 0.00-0.39 poor, 0.40-0.59 fair, 0.60-0.74 good, and 0.75-1.00 excellent [30]. In the present study, a two-way mixed model using absolute agreement between sessions was used to calculate ICC. This is a conservative approach since a prerequisite is that no systematic differences exist between sessions. CV was chosen to give another view on relative reliability and details of the equation used to calculate this have previously been reported [31]. TE is an absolute measure and measures within-subject variation in RT. In this study, TE was calculated using the following equation:

$$ \mathrm{Typical}\ \mathrm{error}=\mathrm{Standard}\ \mathrm{deviatio}{\mathrm{n}}_{diff}/\sqrt{2} $$

The selected sample size in each group was based on recommendations from the COSMIN checklist [32] and experts within the field of reliability studies, which recommend around 20–50 subjects for test-retest studies [33, 34].

### Ethics

Prior to any tests, participants received a written and oral presentation of the experiment by the investigator and gave their written informed consent. The Danish North Jutland regional ethical committee approved the study (N-230878).

## Results

### Upper extremity test

**Fig. 3:**
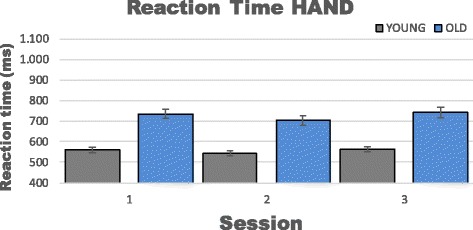
Shows means and 95 % confidence intervals for reaction time (10 recordings) in the upper extremity test (HAND)

Results from the mixed effect model based on 10 trials at each session (1, 2 and 3) indicated a statistical significant effect of age, favoring the young group in RT for the upper extremity (*p* < 0.001; −170.7 ms (95%CI −209.4 to −132.0)). In addition, the mixed model confirmed that no significant learning effects were observed between any of the sessions in the RT test for the upper extremity in either group (Table 2 & Fig. 3).

**Table 2:** Learning effect HAND

Group	Old		Young	
Time	Mean diff (ms)	*P*-value	Mean diff (ms)	*P*-value
1 vs. 2	−32.3	0.17	−17.1	0.23
2 vs. 3	39.3	0.07	20.4	0.10
1 vs. 3	7.1	0.99	3.4	0.99

Shows the estimated mean difference and p-values for learning effects in the upper extremity RT test

Within-day for the upper extremities the lowest ICC value seen was .437 and the highest was .841 for the older adults and for the younger adults the lowest ICC value was .775 and the highest .927. Between-day the lowest ICC value was .633 and the highest .777 for the older adults and for the younger adults the lowest was .786 and the highest .874 (Table 3).

**Table 3:** Reproducibility Hand

OLD							Within-day			Between-day		
	Time 1		Time 2		Time 3		ICC	CV	TE	ICC	CV	TE
Recording	Mean	SD	Mean	SD	Mean	SD	ICC (95 % CI)	(%)	(Absolut)	ICC (95 % CI)	(%)	(Absolut)
1 recording	767.3	±180.4	780.3	±229.5	761.1	±168.0	.437 [0-.767]	23.4	181.4	.745 [.351-.893]	15.4	117.8
Mean of 2	765.0	±171.3	760.9	±179.7	758.4	±145.4	.613 [.117-.822]	17.3	132.3	.633 [.133-.838]	15.7	119.6
Mean of 4	754.5	±150.2	718.6	±146.8	765.5	±174.7	.805 [.584-.916]	11.3	83.3	.757 [.420-.891]	13.7	104.4
Mean of 6	743.9	±135.6	711.8	±147.8	751.8	±157.2	.811 [.594-.918]	10.8	78.8	.756 [.418-.891]	12.6	94.1
Mean of 10	736.0	±135.6	705.6	±150.0	744.5	±154.0	.841 [.659-.932]	10.2	73.6	.777 [.468-.900]	12.2	90.4
Fastest of 3	690.2	±152.7	621.6	±131.5	662.3	±121.2	.792 [.644-.929]	11.4	74.7	.760 [.452-.898]	12.9	87.5
Fastest of 6	648.9	±140.1	596.2	±118.8	630.7	±122.7	.827 [.690-.938]	10.3	64.2	.752 [.426-.893]	13.2	84.6
OLD							Within-day			Between-day		
	Time 1		Time 2		Time 3		ICC	CV	TE	ICC	CV	TE
Recording	Mean	SD	Mean	SD	Mean	SD	ICC (95 % CI)	(%)	(Absolut)	ICC (95 % CI)	(%)	(Absolut)
1 recording	569.8	±81.9	553.3	±103.5	574.5	±113.3	.910 [.782-.962]	6.9	38.6	.861 [.663-.939]	8.6	49.3
Mean of 2	577.5	±84.9	547.8	±94.7	575.9	±111.5	.897 [.819-.965]	6.1	34.6	.786 [.490-.905]	10.4	60.1
Mean of 4	573.0	±88.4	546.8	±88.0	573.4	±103.8	.923 [.869-.974]	5.2	29.2	.806 [.546-.912]	9.7	55.7
Mean of 6	565.2	±84.6	543.7	±82.4	569.9	±96.5	.927 [.866-.974]	5.0	27.9	.841 [.627-.928]	8.5	48.2
Mean of 10	561.5	±84.0	544.8	±79.0	566.8	±99.7	.907 [.806-.962]	5.9	32.4	.856 [.663-.935]	8.3	46.9
Fastest of 3	536.6	±92.6	495.8	±105.3	536.6	±97.4	.841 [.720-.946]	9.0	46.5	.874 [.703-.942]	8.5	45.7
Fastest of 6	507.7	±84.7	479.4	±92.8	516.6	±90.9	.775 [.530-.909]	10.5	52.1	.854 [.662-.934]	8.7	44.7

Data are presented as mean ± SD of either one recording or by collapsing several recordings. ICC denotes Intra-class Correlation Coefficient, CV denotes Coefficient of variance and TE denotes Typical Error

### Lower extremity test

**Fig. 4:**
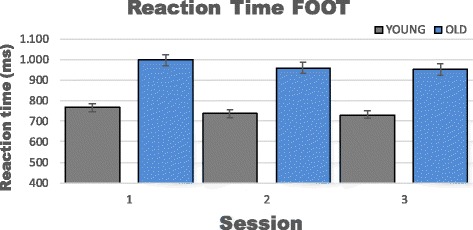
Shows means and 95 % confidence intervals for reaction time (10 recordings) in the lower extremity test (FOOT)

Results based on 10 trials from the mixed effect model indicated a statistical significant effect of age, favoring the young group in reaction time for the lower extremities (*p* < 0.001; −224.3 ms (95%CI −274.6 to −173.9)). In addition, the mixed effect model confirmed that no significant learning effects were observed between any sessions for the older group. This pattern was the same in the younger group between sessions 1–2, 2–3, however between session 1–3 a slight systematic statistical difference was observed (Table 4 & Fig. 4).

**Table 4:** Learning effect FOOT

Group	OLD		YOUNG	
Tiem	Mean diff (ms)	*P*-value	Mean diff (ms)	*P*-value
1 vs. 2	−38.6	0.16	−28.3	0.11
2 vs. 3	6.7	0.99	−7.2	0.99
1 vs. 3	−45.3	0.07	−35.5	0.02

Shows the estimated mean difference and *p*-values for learning effects in the lower extremity RT test

**Table 5:** Reproducibility FOOT

OLD							Within-day			Between-day		
	Time 1		Time 2		Time 3		ICC	CV	TE	ICC	CV	TE
Recording	Mean	SD	Mean	SD	Mean	SD	ICC (95 % CI)	(%)	(Absolut)	ICC (95 % CI)	(%)	(Absolut)
1 recording	1032.8	±252.7	1031.4	±307.0	1004.4	±188.2	.856 [.656-.938]	13.5	139.3	.773 [.480-.903]	13.4	136.2
Mean of 2	1034.6	±224.6	977.5	±227.7	978.4	±183.5	.913 [.834-.967]	8.4	84.1	.793 [.561-.918]	11.6	116.8
Mean of 4	1025.1	±215.5	961.3	±213.6	956.5	±160.1	.911 [.845-.969]	7.8	77.5	.844 [.715-.947]	9.0	89.0
Mean of 6	1016.1	±211.2	958.2	±195.7	960.9	±154.4	.908 [.832-.966]	7.7	76.1	.856 [.721-.948]	8.7	86.4
Mean of 10	997.1	±203.2	958.8	±186.4	952.1	±154.4	.922 [.842-.968]	7.3	71.0	.868 [.742-.952]	8.3	80.7
Fastest of 3	933.7	±213.0	868.2	±192.8	867.6	±145.0	.865 [.746-.949]	10.2	91.8	.800 [.636-.932]	10.6	95.1
Fastest of 6	894.8	±215.0	816.1	±186.2	817.3	±125.0	.837 [.708-.941]	11.3	96.8	.743 [.524-.911]	12.1	103.9
OLD							Within-day			Between-day		
	Time 1		Time 2		Time 3		ICC	CV	TE	ICC	CV	TE
Recording	Mean	SD	Mean	SD	Mean	SD	ICC (95 % CI)	(%)	(Absolut)	ICC (95 % CI)	(%)	(Absolut)
1 recording	825.6	±150.1	776.1	±162.5	755.1	±150.8	.349 [0-.726]	16.4	131.0	.370 [0-.744]	15.9	125.8
Mean of 2	811.2	±122.9	764.4	±156.8	745.4	±135.1	.588 [.095-.824]	13.5	106.3	.568 [.116-.828]	12.4	96.7
Mean of 4	795.1	±126.0	754.5	±148.9	737.6	±124.2	.884 [.776-.957]	7.5	58.4	.789 [.620-.926]	8.7	67.0
Mean of 6	778.3	±115.6	747.6	±148.9	738.6	±121.1	.923 [.849-.971]	6.2	47.0	.850 [.710-.944]	7.4	56.3
Mean of 10	767.8	±115.9	737.9	±140.3	731.0	±122.8	.919 [.840-.969]	6.2	46.7	.870 [.748-.951]	7.1	53.4
Fastest of 3	724.3	±103.8	681.9	±142.4	686.5	±117.7	.785 [.560-.915]	10.1	71.0	.803 [.601-.923]	8.6	60.7
Fastest of 6	674.6	±89.0	650.4	±118.2	664.5	±112.5	.914 [.829-.967]	5.9	39.2	.893 [.753-.952]	6.7	44.9

Data are presented as mean ± SD of either one recording or by collapsing several recordings. ICC denotes Intra-class Correlation Coefficient, CV denotes Coefficient of variance and TE denotes Typical Error

Within-day for the lower extremities the lowest ICC value was .837 and the highest .992 for the older adults and for the younger adults the lowest ICC value was .349 and the highest was .923. Between-day the lowest ICC value was .743 and the highest .868 for the older adults and for the younger adults the lowest was.370 and the highest .893 (Table 5).

## Discussion

The NWB could have potential to become a good clinical tool for assessment of RT in a wide range of populations, as it is inexpensive, portable, and reliable. Overall, the results indicate that the RT tests can differentiate older adults from younger adults. Secondly, that no learning effect within- and between day could be observed for any of the tests with exception of the lower extremity RT test between session 1 and 3 for the younger participants. Finally, that a good reproducibility could be achieved within- and between day for both the upper and lower extremity test using the fastest of three trials in both the older and younger participants.

This was the first study to explore RT measured with standard NWB using custom software. Previously, researchers have primarily measured RT using expensive laboratory equipment [8, 16–20]. The results from the current study showed that for both the upper and lower extremity test the younger adults were markedly faster on average than the older adults (−170.7 ms ~24 % difference and −224.3 ms ~23 % difference, respectively). This was anticipated and not a surprise as numerous studies have shown this previously [16, 21–23].

The mixed effect model used in the present study showed that there was no systematic effect comparing any of the sessions for the RT test in both the upper and lower extremities for both the older and younger adults. The only exception was in the lower extremity test where a significant decrease (−35,5 ms; 4,6 %) in RT was observed for the younger adults between session 1 and 3. However, in the older group a similar trend was seen between session 1 and 3 in the lower extremity test. This possible learning effect might be related to the nature of the lower extremity test, which is weight Bearing contrary to the upper extremity test. If the visual cue i.e. was presented on the left side of the computer screen and the participant was predominantly or fully weight supported on the equivalent leg (left leg) then the RT would be slower compared to if the participant had an equivalent weight distribution on the legs. If this was the case in session 1 and 2 then the participant would have to shift their weight i.e. onto the right leg in order to react appropriately to the visual stimulus (left side). In session 3 the younger adults may have adapted towards this by having a more equivalent weight distribution on their legs resulting in a faster RT compared to session 1. In the future this medio lateral swaying may be avoidable by giving clear and very specific instructions on having their weight equally distributed on their legs. Another possible explanation for the trend between session 1 and 3 in the older group might be that older adults tend to improve speed, accuracy, and consistency of their motor response between sessions [35, 36]. In support of the above and in the present study, the standard deviation in general became smaller with increased number of recordings and across sessions (time 1, 2, and 3) for both groups in the lower extremity test. This underpins that a learning effect across sessions has taken place in the lower extremity test, and that future test protocols should focus on this problem.

In the present study, a good reproducibility (ICC, CV and TE) was achieved within- and between day for both the upper and lower extremity RT test in both groups. However, in 2013 Spiteri *et al.* [20] reported slightly better Coefficient of Variation (CV) values ranging from 1.48 to 3.35 % but similar or lower ICC values ranging from .71 to .83 and for a simple lower extremity RT test in 5 young adults (University sports science students) when averaging 10 trials compared to the present study. These slightly better CV values compared to the current study might be explained by Spiteri *et al.* handpicking 5 out of the original 30 participants for the sub study on reproducibility. Moreover, participants were allowed two practice trials prior to each test before commencing the counting tests. In the current study, this type of participant selection did not occur and not surprisingly, we found slightly higher CV percentage values for both the young (6.2 %) and older (7.3 %) adults. However, the present study produced similar or higher ICC values compared to Spiteri *et al.* when averaging 10 trials. These similar or higher ICC values might be explained by the current study consisted of a much more heterogeneous study-population in both groups than the very small study-population in the Spiteri *et al.* study. In another reproducibility study Mercer and coworkers [15] evaluated a simple, inexpensive, and portable ruler-method for measuring RT on 30 community-dwelling older adults using an intra-day (20 min pause) design. They found the method to be outside of an acceptable reproducibility range as ICC was .53 between sessions. In addition, they observed a significant learning effect between sessions, which further disqualified the method. However, the Mercer study did only report ICC values with respect to reproducibility and this may have played to their disadvantage, as other statistical methods (which are effected to a minor degree by the study-population) could have shown greater reproducibility. The ICC calculation is depends on the ratio of the variability between participants to the total variability, and is thus affected by factors related to the study sample itself. The CV percentage or similar (Bland-Altman plots, Limits of Agreement) are less effected by study sample and are important to report as they will give an indication either in percent or absolute values to the measurement error of the method [29]. This becomes of great importance when evaluating the effect of an intervention study or a rehabilitation course, as some of the potential effect achieved may/should be attribute to measurement error. Based on the measures of reliability (ICC) and agreement (CV, TE) the authors recommend using the fastest trial of three in both the upper and lower extremity RT test in both groups in order to minimize measurement error and at the same time be time efficient. However, to avoid a learning effect across days of testing it is recommended that habitation trials are applied for both young and old adults when testing RT in the lower extremities.

The strength of the current study is the availability of the NWB’s. Presently approximately 43 million Wii-boards have been sold across the world. In addition, it is a very cost-efficient and portable method compared to existing methods. Moreover, we were able to test both upper and lower extremities, and explore within- and between day reproducibility in one study. Finally, the present study concurred with internationally accepted guidelines in terms of reporting reproducibility studies as several measures of both reliability (ICC) and agreement (CV and TE) were reported [29]. A weakness in the current study is the lack of validation. This study did not correlate results with a ‘gold standard’ within reaction time testing, which would have added to the potential future use of the test in clinical settings. Finally, the custom software prepared for the current study is not yet widely available limiting the usefulness of the study. However, in the near future the authors plan to validate the Wii-RT test against a ‘gold standard’ method and make the software widely available to clinicians and researchers.

## Conclusion

This study found that a portable reaction test utilizing a standard Nintendo Wii board could differentiate between young and older adults in upper and lower extremities. In addition, no systematic significant differences were observed within-day or between-day for the reactions tests with exception of the lower extremity test between session one and three in the young group. A good reproducibility was observed in both the upper and lower extremity test for both the young and old group using the fastest of the three recordings. Future studies should aim at validating the Wii-RT test against a “gold standard” reaction test.
